# Insight into the Sorption of 5-Fluorouracil and Methotrexate onto Soil–pH, Ionic Strength, and Co-Contaminant Influence

**DOI:** 10.3390/molecules26061674

**Published:** 2021-03-17

**Authors:** Katarzyna Markiewicz, Anna Białk-Bielińska, Paulina Łukaszewicz, Piotr Stepnowski, Joanna Dołżonek

**Affiliations:** Department of Environmental Analysis, Faculty of Chemistry, University of Gdansk, ul. Wita Stwosza 63, PL-80-308 Gdańsk, Poland; katarzyna.markiewicz@ug.edu.pl (K.M.); paulina.lukaszewicz@ug.edu.pl (P.Ł.); piotr.stepnowski@ug.edu.pl (P.S.); joanna.dolzonek@ug.edu.pl (J.D.)

**Keywords:** anticancer drugs (ADs), cytostatic drugs, environmental fate, leaching test, mobility

## Abstract

Nowadays anticancer drugs (ADs), like other pharmaceuticals, are recognized as new emerging pollutants, meaning that they are not commonly monitored in the environment; however, they have great potential to enter the environment and cause adverse effects there. The current scientific literature highlights the problem of their presence in the aquatic environment by publishing more and more results on their analytics and ecotoxicological evaluation. In order to properly assess the risk associated with the presence of ADs in the environment, it is also necessary to investigate the processes that are important in understanding the environmental fate of these compounds. However, the state of knowledge on mobility of ADs in the environment is still very limited. Therefore, the main aim of our study was to investigate the sorption potential of two anticancer drugs, 5-fluorouracil (5-FU) and methotrexate (MTX), onto different soils. Special attention was paid to the determination of the influence of pH and ionic strength as well as presence of co-contaminants (cadmium (Cd^2+^) and another pharmaceutical—metoprolol (MET)) on the sorption of 5-FU and MTX onto soil. The obtained distribution coefficient values (*K_d_*) ranged from 2.52 to 6.36 L·kg^−1^ and from 6.79 to 12.94 L·kg^−1^ for 5-FU and MTX, respectively. Investigated compounds may be classified as slightly or low mobile in the soil matrix (depending on soil). 5-FU may be recognized as more mobile in comparison to MET. It was proved that presence of other soil contaminants may strongly influence their mobility in soil structures. The investigated co-contaminant (MET) caused around 25-fold increased sorption of 5-FU, whereas diminished sorption of MTX. Moreover, the influence of environmental conditions such as pH and ionic strength on their sorption has been clearly demonstrated.

## 1. Introduction

Cancer is a serious public problem and the most common cause of death worldwide [1,2]. According to the World Health Organization (WHO), only in 2018, the estimated number of new cases of cancer amounted to about 18 million, of which human mortality was at the level of 9.6 million deaths [3]. By 2030, the disease and mortality rates are expected to increase to about 22 million and about 13 million deaths, respectively [1,4].

An inherent consequence of the increase in cancer incidence is the constant search for new, effective solutions, the increase in the consumption of anticancer drugs (ADs) [3,5], and the risk of introducing more of these substances into natural ecosystems. These drugs, due to their non-specific mode of action, as well as mutagenic, carcinogenic, cyto- and genotoxic properties, disrupting the functioning of the endocrine system in eukaryotic cells, should be given greater and special attention because information on their potential risk to both humans and the environment is still limited [3,6,7].

Nowadays, ADs, like other pharmaceuticals, are recognized as new emerging pollutants, meaning that they are not commonly monitored in the environment; however, they have great potential to enter the environment and cause adverse effects there [8]. Moreover, after administration, they are not fully metabolized; hence, a significant part of them is excreted in unchanged and metabolite forms [9]. Low biodegradability of ADs, combined with incomplete removal of these compounds in wastewater treatment plants, results in their release to the environment [7,10]. The first reports confirming the presence of these drugs in aquatic ecosystems appeared in the 1980s [3,7]. The occurrence has been confirmed not only in hospital and municipal wastewaters (recognized as the main source of ADs emissions to the environment [10,11,12]) but also in surface waters at the level of ng L^−1^ concentrations [13,14,15,16]. Even though their environmental concentrations are quite low, their high biological activity may lead to undesirable effects due to their inherent cytostatic and cytotoxic properties [3,17,18]. The current scientific literature signals the problem of their residues getting into the aquatic environment, presents the results of analytical and ecotoxicological studies [10,11,15,19,20], as well as distinguishes the priority drugs in terms of environmental monitoring, which is still not obligatory.

In order to properly assess the risk associated with the presence of ADs in the environment, it is also necessary to investigate the processes that are important in understanding the environmental fate of these compounds. Taking into account the fact that some drugs present in the wastewater have the ability to bind to the sludge [7,21], which is then used to fertilize soils, it becomes essential to pay attention to the sorption onto soil as well. It is an important process controlling the transport of organic compounds in the environment, which may also significantly change chemical reactivity of pollutants. However, the state of knowledge on mobility of ADs in the environment is still very limited. For example, Azuma et al. (2017) and Azuma (2018) have presented the determined log*K_d_* values of the selected anticancer drugs (bicalutamide, tamoxifen, cyclophospohamide, capecitabine, doxifluridine, tegafur) in river sediments [22,23]. Obtained *K_d_* values (0.36–398 L·kg^−1^) show the wide variety in mobility of anticancer drugs in terrestrial compartments such soil or sediment, indicating the need for examination of other compounds belonging to this group of pharmaceuticals. The sorption of uncharged organic chemicals onto natural sorbents is mainly dominated by “hydrophobic interactions”. Such sorption mechanism can be considered as independent of pH changes, and hence predicting mobility of non-polar and uncharged compounds is quite a straightforward process and might be predicted based on logK_OW_ values of these chemicals. However, in the case of ionizable compounds, differences in sorption potential to various soils and sediments as well as the influence of pH and ionic strength on this phenomenon is evident [24]. For example, Tolls et al. showed that sorption of ionizable chemicals onto soil displays a wide range of mobility (0.2 < *K_d_* < 6000 L·kg^−1^) [25]. To date, the attention has been paid mostly on external factors such as pH, ionic strength, or temperature. Nevertheless, pharmaceuticals do not occur alone in the environment; therefore, the influence of co-existing substances should also be taken into consideration in the comprehensive evaluation of environmental fate of pharmaceuticals. It has been already shown in the literature that the presence of other contaminants affects the environmental fate of pharmaceutical residues. For example, Chun et al. (2005) proved that co-occurring antibiotics affect persistence and transformation of hormones. It has also been demonstrated that the presence of three co-compounds (veterinary antibiotics) improves the stability of 17β-estradiol in soil [26]. On the other hand, Srinivasan et al. (2013) showed that the presence of hormones affects sorption of sulphonamides, leading to decrease in sorption potential changing the mechanism of sorption [27]. In our previous research we showed that metoprolol as co-contaminant may lower the mobility of cyclophosphamide and ifosfamide in soil [28]. As these compounds are present in different ionic species, depending on the pH, their mobility in the soil compartments can be modulated by the changes of pH or ionic strength.

Therefore, the main aim of our study was to investigate the sorption potential of two anticancer drugs: 5-fluorouracil (5-FU) and methotrexate (MTX) (Table 1) onto different soils using the batch equilibrium method (Organization for Economic Cooperation and Development, OECD 106). Pharmaceuticals selected for this study have been placed on the list of “Cancer drugs approved by the Food and Drug Administration (FDA) for breast cancer”, which makes them frequently used agents in anticancer therapy [13]. However, they are not included in any monitoring programs, especially with regard to the quality of wastewater or purified tap waters [29,30,31]. Additionally, it was also proved that these compounds pose not only strong cytotoxic activity but also estrogenic activity [7], which can affect non-target organisms living in the environment. For example, high toxicity has been already proven for 5-FU in chronic test towards *Daphnia magna* (EC_50_ 26.4 µg·L^−1^) [15]. Hence, the assessment of their presence and fate in the environment is of the utmost importance. No information about their sorption potential to soil is available in the literature. Only some studies referring to their sorption ability to microplastics [32] or carbon nanotubes [33,34] have been recently undertaken.

Special attention was paid to the determination of the influence of pH and ionic strength as well as the presence of co-contaminant on the sorption of 5-FU and MTX onto soil. As an exemplary co-contaminant, another pharmaceutical commonly detected in different environmental compartments—metoprolol (MET, beta-adrenergic blocking agent)—was selected, as well as cadmium (Cd^2+^)—as a representative of heavy metals well-known as persistent pollutants.

## 2. Results and Discussion

### 2.1. The Assessment of the Sorption Potential of 5-FU and MTX onto Soils

**Figure 1:**
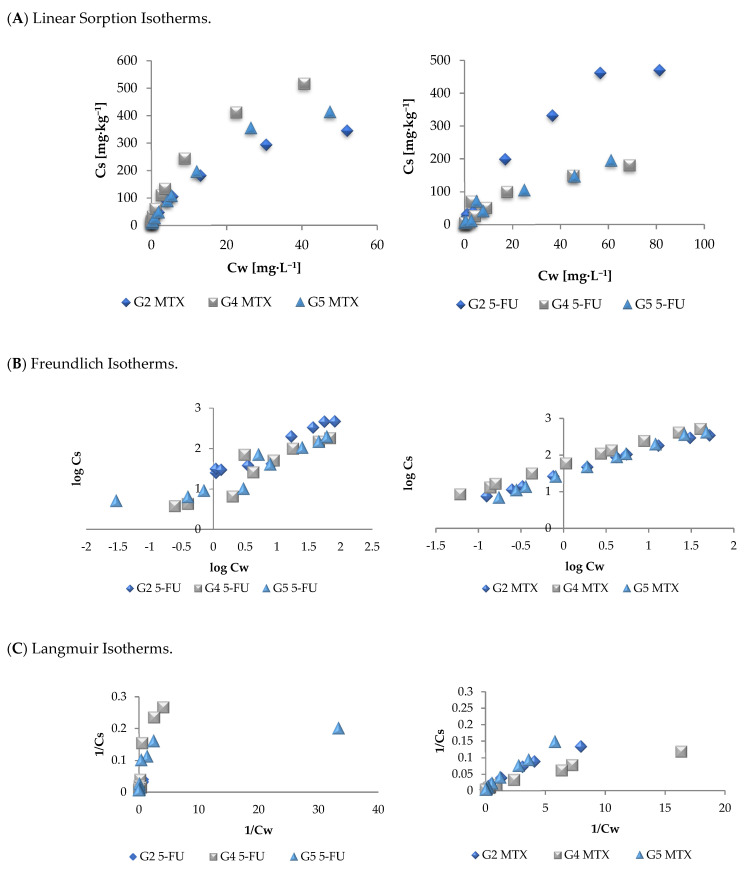
Selected 5-FU and MTX sorption isotherms (Basic conditions: medium: 0.01 M CaCl_2_; concentration range of ADs: from 0.625 to 100 mg·L^−1^**;** adsorption equilibrium time: 24 h; optimal soil−to−liquid (S/L) ratio: 5-FU (G2 soil = 1:25; G4 and G5 soil = 1:5); MTX (G2, G4, G5 soil = 1:15).

The determined selected sorption isotherms of 5-FU and MTX onto investigated soils are presented in Figure 1. The parameters of all the isotherms are presented in Table 2.

The *K_d_* values were determined based on linear sorption isotherm (Equation (1) in the Materials and Methods section). The obtained *K_d_* values ranged from 2.52 to 6.36 L·kg^−1^ and from 6.79 to 12.94 L·kg^−1^ for 5-FU and MTX, respectively (Table 2).

The *K_d_* values for pharmaceutical substances presented in the literature are characterized by considerable diversification, which is closely related to the type and nature of the solid matrix. For example, for veterinary drugs from the group of imidazoles, phenicols, beta-blockers, or sulfonamides, the ranges of the sorption coefficients are 0.5–0.7 L·kg^−1^ [37], 0.2–8 L·kg^−1^ [38], 9.3–37.6 L·kg^−1^ [39], and 0.6–206 L·kg^−1^ [38]. For comparison, the *K_d_* values for veterinary antibacterial drugs from the tetracycline and fluoroquinolone groups are much higher and are in the following ranges: from 420 to 2386 L·kg^−1^ [38,39] and from 260 to 6310 L·kg^−1^ [39]. Such high *K_d_* values indicate the unlikely mobility of these compounds. The tendency to strong binding to the surface of solid components is in this case related to the ability to form stable complexes with double-charged cations present in this type of matrix [40]. However, it should be emphasized that it has been proven that even substances showing a high tendency to sorption do not have to remain immobilized in the solid components and may migrate to waters [41]. Although sorption coefficients (*K_d_*) of 5-FU and MTX are respectively low, according to categorization presented in the literature, the mobility of both investigated compounds in soil may be considered as low or slight [42].

**Figure 2:**
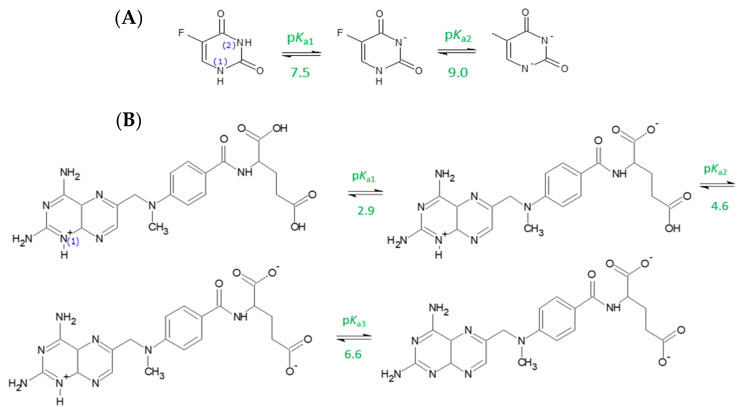
Acid–base equilibrium of 5-FU (**A**) and MTX (**B**) [43].

Based on our results it can be concluded that MTX has slightly stronger sorption potential than 5-FU; however, different correlations have been observed of the investigated compounds and soil properties. In case of 5-FU, the highest sorption potential was observed in case of soil characterized by the highest organic matter (OM) content (G2–alluvial soil). In the case of ionic compounds, it is crucial in the evaluation of their sorption potential to take into account their acid–base properties, which for the selected anticancer drugs have been determined and discussed in detail in our previous paper [43]. The proposed acid–base equilibria of these drugs depending on pH are presented in Figure 2 [43].

Briefly, two pKa values of 5-FU (pK_a1_ = 7.53, pK_a2_ = 9.01) lead to two steps of dissociation. We have observed that at pH ~6, 5-FU starts to dissociate to monoanionic species. At pH equal to pK_a1_, 50% of 5-FU molecules exist as neutral species, whereas 50% exist as monoanionic molecules. The dianionic species of 5-FU is formed at the pH that corresponds to pK_a2_ (9.01); however, above a pH of 9.5, the dianionic form is predominant [43]. Based on these findings, as well as according to Wileńska et al. (2019), it might be assumed that during the sorption experiments 5-FU occurred mostly as neutral species. Thus, its sorption onto soil can be revealed from partitioning to neutral moieties of organic matter by, for example, π–π interactions.

In the case of MTX (pK_a_: 2.91, 4.64, 6.57), at pH below the value of 2.91, MTX exists as an organic cation (Figure 2). Between pH 2.91 and 4.64, the nitrogen atom is still protonated, whereas the γ-carboxyl group is deprotonated (net neutral charge). Above the pH 4.64, the second carboxyl group is deprotonated (monoanionic form), whereas the nitrogen atom is deprotonated in solution of pH above 6.57. Hence, at a pH above 6.5, the dianionic form dominates [43].

The highest *K_d_* for MTX (12.94 L·kg^−1^) was determined for soil G4 (acid brown soil) characterized by highest content of mineral fraction, moderate content of organic matter (OM = 7.7%), and lowest pH (pH_KCl_ = 5.1) among tested soils (Table 1).

Taking into consideration the pH of the water phase during experiments (7.1, 5.1, 5.8, respectively, for soil G2, G4, G5), it might be assumed that in the case of soil, G2 and G5 MTX occurred mostly as molecules possessing one positive charge (protonated nitrogen in pteridine ring) and, due to dissociation of carboxylic groups, two negative charges. In the case of soil G4, approximately 50% of MTX molecules had one carboxylic group not dissociated (zwitterionic species), and the rest occurred as monoanionic species. Therefore, it might be suspected that MTX could interact with soil via electrostatic interactions due to both positive and negative charge via cation–anion interactions or cation bridging.

Taking into consideration the values of the correlation coefficients (*R*^2^) of the determined sorption isotherms (Table 2), the highest values were observed for Freundlich isotherms, which proves that this isotherm can be used for the description of the sorption process of these pharmaceuticals onto soil. The values of 1/n of the Freundlich isotherm, describing relative bulk and diversity of energies associated with sorption process, in the case of MTX are in the range from 0.65 (MTX, G2) to 0.75 (MTX, G5) and from 0.54 (5-FU, G5) to 0.73 (5-FU, G4), which represents a convex, curved downward isotherm type, where the marginal sorption energy decreases with increasing concentration of sorbate [24]. It demonstrates that sorption of MTX and 5-FU onto all soils decreases with an increasing amount of the compound. The higher values of K_F_ obtained for MTX than for 5-FU correspond well with previously discussed *K_D_* values and reflect higher sorption potential of MTX than 5-FU. On the other hand, the R^2^ values for Langmuir isotherms were in most cases lower than for Freundlich isotherm, proving that this model is less suitable to describe sorption process of investigated anticancer drugs.

Furthermore, correlation coefficients determined for Dubinin–Raduszkiewicz (D-R) and Temkin isotherms also indicates that these models do not describe this phenomenon well (*R*^2^ in the range of 0.513–0.903 and 0.648–0.856, respectively). The D-R isotherm model assumes that the porous structure of the sorbent is responsible for sorption. In the case of the tested soils, the G5 soil with the highest specific surface area (SA = 10.9 m^2^·g^−1^) may be characterized by high porosity, which in turn may be a factor enhancing the sorption of low-molecular compounds. The *B_D_* is the coefficient describing the adsorption energy, which also gives an overview of the average free energy *E_D_* [kJ·mol^−1^] [44] and the resulting nature of the responsible interactions for sorption. In general, it is assumed that if *E_D_* values are in the range of 8–16 kJ·mol^−1^, sorption is based on the ion exchange process. In our study, the determined *E_D_* for all the tested analytes did not exceed the value of 2.61 kJ·mol^−1^, which indicates that physical sorption of MTX and 5-FU occurs onto the investigated soils [36,45]. It may be concluded that selected drugs will be retained in soil structures as a result of weak van der Waals interactions due to the chemical form in which they occur (uncharged molecule). Only the case of MTX ion exchange could be considered. However, it was not confirmed by the discussed D-R model. Therefore, the more probable mechanism seems to be cation bridging, based on the interaction of negatively charged carboxyl groups of MTX (at pH > 6.5) with cations (e.g., Ca^2+^) attached to the negatively charged active centers of soil colloids [46].

Finally, the equilibrium binding constant (*K*_0_) related to the maximum binding energy in the Temkin isotherm was up to 5.59 L·mg^−1^, which confirms the low sorption potential of the selected anticancer drugs. The obtained positive values of the ∆*Q* indicate the exothermic nature of sorption of the tested anticancer drugs. In addition, the determined B coefficient, which is related to the adsorption energy, is lower than 20 J·mol^−1^, which also confirms physical adsorption process of those compounds [45].

### 2.2. The Influence of pH

**Figure 3:**
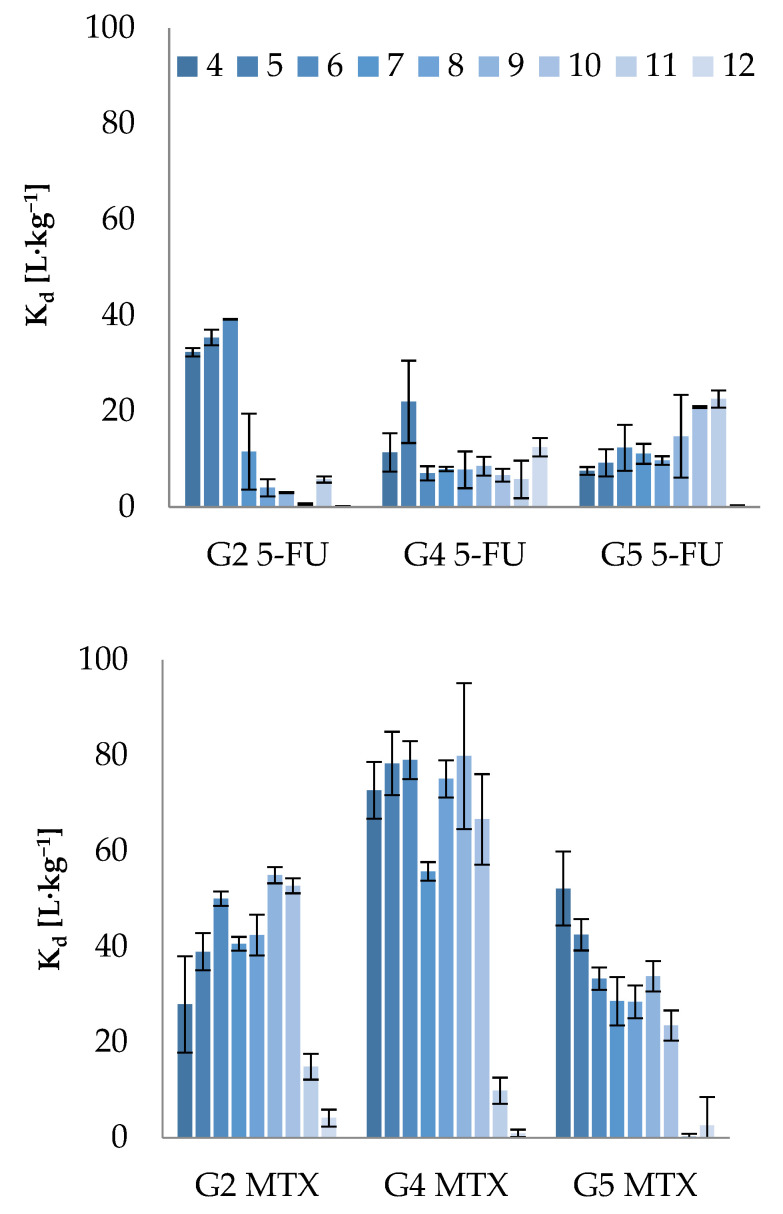
The influence of pH on the sorption potential of 5-FU and MTX (Basic conditions: medium: 0.01 M CaCl_2_; pH range from 4 to 12; concentration level of ADs: 5 µg·mL^−1^; adsorption equilibrium time: 24 h; optimal S/L ratio: 5-FU (G2 soil = 1:25; G4 and G5 soil = 1:5); MTX (G2, G4, G5 soil = 1:15).

The *K_d_* for 5-FU and MTX determined in different pH of liquid phase are presented in Figure 3.

It was observed that along with the increase of the concentration of monoanionic form of 5-FU (the anionic species of 5-FU occurs at pH above 7.53 [43])**,** its sorption potential onto G2 soil (with the highest OM content) decreased; however, it did not change significantly for soils G4 and G5 (Figure 3). It can be explained by the fact that along with increasing pH, the anionic form of 5-FU was repulsed by the negatively charged surface of G2 soil, which is in agreement with the value of CEC (cation exchange capacity) of this soil, reflecting negative charge abundance on the soil surface—increasing with raising pH. It was already reported that the sorption of pharmaceuticals, which occurs as organic anions or zwitterions, e.g., sulfachloropyridazine, tylosin, and oxytetracycline, decrease with the increase of pH [47]. On the other hand, Kovalova et al. observed the same trend (decreased sorption at increased pH) on positively charged powdered activated carbon, which was probably related to higher solubility [48]. Hence it cannot be excluded that decreased adsorption of 5-FU onto soils at higher pH is also attributed to its better solubility.

The negligible influence of pH on sorption potential of 5-FU was observed in case of soil G4, confirming thereby OM content as the key factor responsible for sorption of this chemical onto soils. However, for soil G5, characterized with the highest surface area, the influence of pH was observed only at very alkaline solution (pH 12), which might be attributed to increased negative charge on the mineral fraction of soil.

Strong dependence of sorption potential on pH and hence the ionization form of the analyte was also observed for MTX (Figure 3). Lower sorption and hence increased mobility of this compound was observed with the increase of pH, which can be explained by the presence of different ionic species (Figure 2). When pH increases, the sorption is decreased, with significant drop at pH 11, at which MTX occurs in the solution as an anionic species at 100% due to deprotonation of both carboxyl groups. Hence, a decrease in sorption may arise from electrostatic repulsion between the negatively charged species of this compound and negatively charged soil surface, which indicates that ionic interactions are involved in the sorption mechanism of MTX.

### 2.3. The Effect of Ionic Strength

**Figure 4:**
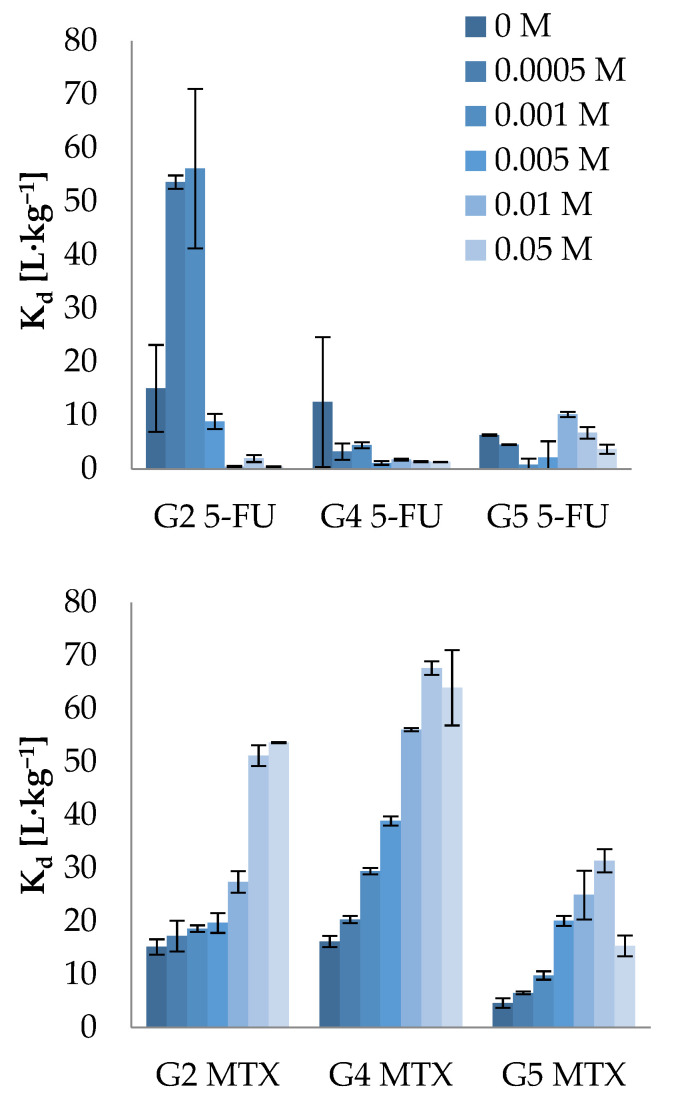
The influence of ionic strength on the sorption potential of 5-FU and MTX (Basic conditions: concentration range of CaCl_2_: from 0, to 0.1 M; concentration level of ADs: 5 µg·mL^−1^; adsorption equilibrium time: 24 h; optimal S/L ratio: 5-FU (G2 soil = 1:25; G4 and G5 soil = 1:5); MTX (G2, G4, G5 soil = 1:15).

The influence of ionic strength on the sorption potential of investigated pharmaceuticals is presented in Figure 4.

In the case of 5-FU, the increase of ionic strength caused the decrease of sorption potential onto soils in the following order: G2 > G4 > G5; hence, the strongest dependency was observed for soil with the highest OM content (18.64%), whereas there was negligible influence for soil with the lowest OM (4.13%) among investigated soils, which tallies with the previously mentioned hypothesis about high affinity of 5-FU to soil organic matter.

On the other hand, a strong increase of the sorption potential of MTX was observed with the increase of ionic strength for all tested soils. The same trend has been previously reported in the literature for acidic compounds (negatively charged at experimental conditions), e.g., sulfonamides [47,49], pesticides [50], or Brilliant Blue FCF [51]. One of the possible explanations may be the compensation of the electrical double layer, leading to the reduction of electrostatic repulsion of the MTX molecule with negatively charged soil surface, lowering the pH of the solution as a result of the exchange of hydrogen (H^+^) and aluminum ions (Al^3+^) by Ca^2+^ and a slight shift of the acid–base equilibrium toward a positively charged form of MTX. Moreover, as MTX occurs as zwitterion at pH close to 7, increased sorption could arise from cation bridging, which is probable in the presence of a high concentration of Ca^2+^. Therefore, our investigation also proved that the type of interaction and hence the sorption mechanism of MTX may change significantly depending on environmental conditions such as salinity.

### 2.4. The Impact of the Presence of Co-Contaminant on the Sorption of Selected Anticancer Drugs onto Soils

#### 2.4.1. The Influence of Metal Presence (Cd^2+^)

As heavy metal cations interact with the soil surface mainly through ion exchange and surface complexation to form metal-carbonate bonds [52], it is possible that active sites of the sorbent will be blocked and/or change, which may change the mobility of different chemicals in the soil structures. On the other hand, it is also possible that the adsorbed heavy metals may also constitute new active sites and lead to the decreased mobility of chemicals. This may also affect the sorption potential of the selected in our study anticancer drugs. For all these reasons, the influence of the presence of cadmium in the soil on the sorption of selected anticancer drugs in our study was investigated.

Cadmium was selected as a representative of divalent metals. Soil contamination with cadmium is a ubiquitous environmental problem mainly related to intensive agricultural practices. The use of animal manure, phosphorus fertilizers, or sewage sludge, in which this metal is commonly found, results in its presence and accumulation in soil environment [34,53]. Therefore, cadmium can influence the behavior of other contaminants in soils and was selected in our study. Moreover, cadmium contamination of soils in Poland is monitored in accordance with the Ordinance of the Minister of the Environment of 9 September 2002 (Dz.U. 2002 nr 165 poz. 1359) [54].

**Figure 5:**
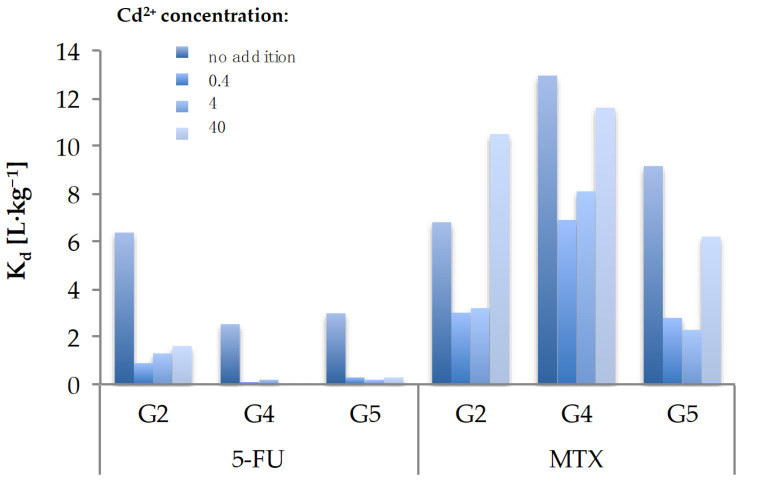
The influence of Cd^2+^ presence on the sorption potential of 5-FU and MTX (Basic conditions: medium: 0.01 M CaCl_2_; concentration levels of cadmium: 0.4, 4.0, 40 mg·kg^−1^ d.m.; adsorption equilibrium time: 24 h; optimal S/L ratio: 5-FU (G2 soil = 1:25; G4 and G5 soil = 1:5); MTX (G2, G4, G5 soil = 1:15).

The experiments were carried out for three different concentrations of Cd^2+^ in the soil. The determined *K_d_* values for the selected drugs are presented in the Figure 5.

As it can be observed in the case of 5-FU, its sorption decreased in the case of soil contaminated with cadmium in relation to systems without this pollutant. However, for MTX, it was noticed that the *K_d_* parameter increased with the increase in the concentration of cadmium in the soil. This phenomenon may indicate the formation of a cationic bridge by cadmium ions attached to the negatively charged soil surface with MTX, which is negatively charged as a result of dissociation of carboxyl groups. It hereby confirms our previous conclusions on the possible mechanism of MTX sorption in the soil environment.

#### 2.4.2. The Influence of the Presence of Metoprolol

Metoprolol (MET)—a pharmaceutical commonly applied in treating heart diseases and hence commonly detected in various environmental samples [36,55]—was selected as the representative compound for the investigation of the influence of sorption of MTX and 5-FU onto selected soils. The single point sorption coefficients (K_d_*) determined for this purpose are presented in Table 3.

It was observed that in almost all cases the sorption potential of MTX in the presence of MET was lower than without the co-contaminant. Taking into consideration the positive charge of the MTX molecule and the positively charged molecule of MET, such an observation may be attributed to competition between those chemicals for the same negatively charged interaction sites on the surface of analyzed soils. However, due to abundance of aromatic rings in both compounds, π–π interactions may also be the source of this competition, especially due to the fact that the strongest difference was observed for soil G2, rich in OM content in comparison to other investigated soils. In order to prove that lower sorption is attributed to MET occurrence and the mentioned competition phenomenon, the sorption of MET was also determined. As the determined K_d_* was lower in the presence of MTX, it clearly confirms our surmise.

In contrast to MTX, the sorption affinity of 5-FU in the presence of the co-contaminant (metoprolol, MET) increased (in comparison to its sorption potential as single sorbate in test system) (Table 3). Moreover, it must also be highlighted that for this soil G2 (high OM content), the simultaneous increase of sorption potential of MET in the presence of 5-FU was observed, which confirms an interaction between 5-FU and MET affecting their sorption mutually. It has been already shown in the literature that sorption of beta-blockers (e.g., MET) depends strongly on organic carbon content [36]. Therefore, increased sorption of 5-FU in the case of this soil is justified. Moreover, sorption of MET also increased when tested in the mixture with 5-FU.

In the case of soil G5 (relatively low OM, high SA), a similar increase of sorption of 5-FU was reported, whereas sorption of MET did not increase and even decreased in the same mixture. Sorption of MET onto soil characterized by high SA occurs probably due to its cationic species; hence, interaction via electrostatic attraction is possible onto mineral fraction of soil. Adsorbed MET molecules might create new adsorption sites for 5-FU, while adsorbed 5-FU is less attractive for MET than the negatively charged soil surface. Such a hypothesis tallies with results obtained for soil G4. Both low SA and relatively low OM did not provide enough attractive sorption sites for 5-FU; hence, there was low probability to increase sorption of MET. Additionally, MET did not increase sorption of 5 FU due to its relatively low sorption potential to this soil (K_d_* = 14.97 L·kg^−1^).

## 3. Materials and Methods

### 3.1. Chemicals

Standards of 5-fluorouracil (5-FU), methotrexate (MTX), metoprolol tartrate (MET) were purchased from Sigma-Aldrich (Steinheim, Germany). Deionized water was produced by the Hydrolab System (Gdańsk, Poland). Acetonitrile (ACN), methanol (MeOH), calcium chloride (CaCl_2_), sodium hydroxide (NaOH) were purchased from POCH-AVANTOR (Gliwice, Poland). Dimethyl sulfoxide (DMSO) was purchased from J. T. Baker (Gross Gerau, Germany). Formic acid (HCOOH) and hydrochloric acid (HCl) were purchased from Chempur (Piekary Śląskie, Poland) and STANLAB (Lublin, Poland), respectively.

### 3.2. Soils

The experiment was carried out using three types of soils characterized by different physicochemical properties (Table 4).

### 3.3. Conceptual Approach

**Figure 6:**
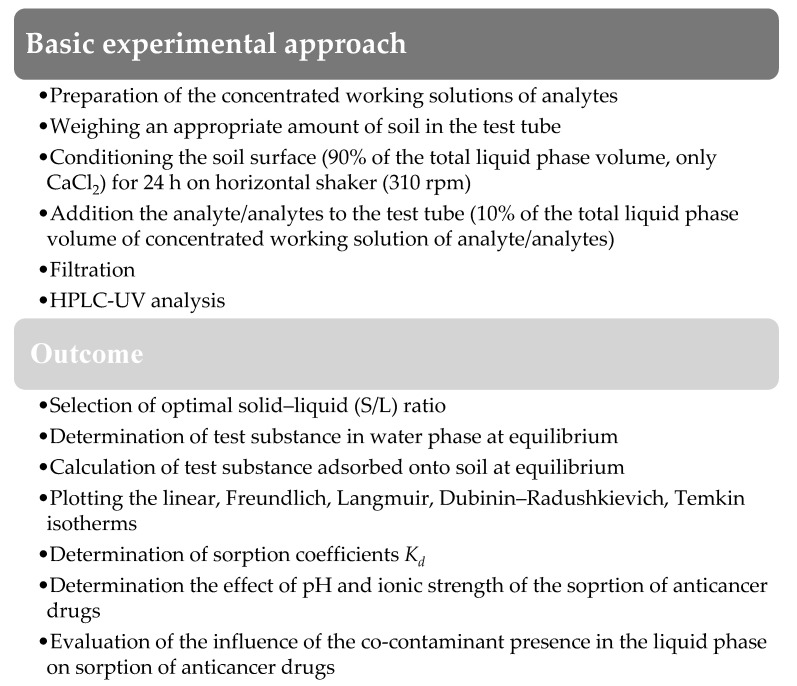
Conceptual approach of the research presented in this study.

The sorption of pharmaceuticals onto selected soils was performed according to the procedure of the Organization for Economic Cooperation and Development (OECD) 106 using the batch equilibrium method [56]. All steps of the experiment performed in this study are shown in Figure 6. At each stage, three types of sample were prepared: test sample (soil, liquid phase, and analyte/s), blank sample (soil and liquid phase), and control sample (analyte/s in the liquid phase).

### 3.4. Conceptual Approach

#### 3.4.1. Solutions Used in the Preliminary Studies and Determination of Sorption Isotherms

The standard stock solution of 5-FU (1000 mg·L^−1^) was prepared in the water solution of 0.01 M CaCl_2._ To select the optimal soil-to-liquid (S/L) ratios and equilibrium time of the experiments, standard stock solution of 5-FU was diluted using 0.01 M CaCl_2_ to the concentration of 10 mg·L^−1^.

The standard stock solution of MTX (1000 mg·L^−1^) was prepared in DMSO_._ To select the optimal S/L ratios and equilibrium time of the further experiments, standard stock solution of MTX was diluted using 0.01 M CaCl_2_ to the concentration of 10 mg·L^−1^ so that the percentage of DMSO in all experiments did not exceed 1%.

To determine sorption isotherms, working stock solutions of 5-FU and MTX were prepared at 10 concentration points in the range from 6.25 to 1000 mg·L^−1^ as serial dilutions using 0.01 M CaCl_2_ as a solvent. In fact, the concentrations of analytes within the tests were 10 times lower (in the range of 0.625–100 mg·L^−1^) than prepared working solutions of analytes, due to 10-fold dilution resulting from previous soil surface equilibration step.

Additionally, it must be also highlighted that all the solutions used in our study contained the sodium azide (NaN_3_) in the concentration of 100 mg·L^−1^ in order to avoid the biodegradation of the investigated anticancer drugs during the performed experiments.

#### 3.4.2. Solutions Used in the Evaluation of the Influence of pH and Ionic Strength

The standard stock solution of 5-FU (1000 mg·L^−1^) was prepared in MeOH. The solutions of 0.01 M CaCl_2_ at proper pH were obtained with addition of 1 M HCl or 1 M NaOH. In order to prepare working solution of 5-FU at concentration of 50 mg·L^−1^, 2.5 mL of stock solution was placed in volumetric flask (50 mL), evaporated to dryness under the stream of nitrogen. Subsequently a volumetric flask was filled up with 0.01 M CaCl_2_ previously adjusted to required pH. Analogically, in order to investigate the influence of ionic strength the solutions at different concentrations of CaCl_2_ (0, 0.0005, 0.001, 0.005, 0.01, 0.05, and 0.1 M) were prepared. The further steps were analogous to the procedure described above.

The solutions of MTX used to investigate the influence of pH and ionic strength were prepared in the similar way. The evaporation step was excluded because the standard stock solution (1000 mg·L^−1^) was prepared in DMSO, and the concentration of working solution of MTX was lower (10 mg·L^−1^) in order not to exceed the amount of 1% of DMSO in the test samples.

### 3.5. Preliminary Studies

In the first stage of our sorption experiment it was necessary to determine the optimal soil-to-liquid (S/L) ratio as well as the adsorption equilibrium time. For this purpose, different options were tested depending on the type of soil and compound. The S/L ratio was tested in five options: 1:2; 1:5; 1:15; 1:25; 1:50, and the adsorption equilibrium time was monitored in the range from 1 h up to 24 h. Finally, the best/optimal conditions in which the adsorption of each of the analytes was at least 20% onto the selected soils (fulfilling the requirements of OECD 106 guideline [56]) are presented in Table 5.

### 3.6. Determination of Distribution (Sorption) Coefficients and Sorption Isotherms

Determination the amount of compound bound to the solid phase at equilibrium state, in the soil/liquid system, was possible by ascertaining the equilibrium and determination the mass concentration of substance in the aqueous phase. Based on this, the sorption isotherms were determined in the concentration range from 0.625 up to 100 mg·L^−1^. The distribution (sorption) coefficient (*K_d_*), expressed as the ratio of the adsorbate concentration to the concentration of the contaminant remaining in the liquid phase, was calculated based on the obtained linear isotherm (Equation (1)).

(1) $$c_{S}=K_{d}\cdot c_{W}$$

where *c_s_* is the content of substance adsorbed onto soil at adsorption equilibrium (mg·kg^−1^); *c_w_* is the mass concentration of substance in the aqueous phase at adsorption equilibrium (mg·L^−1^); and *K_d_* is the distribution coefficient (L·kg^−1^).

The experimental data was subsequently fitted to Freundlich, Langmuir, Dubinin–Radushkievich, and Temkin isotherm. Detailed description of these isotherms is presented in our previous papers [36,49], hence only applied equations have been presented here.

The Freundlich isotherm (Equation (2)):

(2) $$\log c_{S}=1/n\log\cdot c_{W}+\log K_{F}$$

where *c_s_* is the content of test substance adsorbed onto soil at adsorption equilibrium (mg·kg^−1^); *c_w_* is the mass concentration of test substance in the aqueous phase at adsorption equilibrium (mg·L^−1^); *K_F_* is the Freundlich adsorption coefficient (mg^1−1/n^·kg^−1^·L^1/n^); 1/*n* is the Freundlich exponent or linearity factor (a constant depicting the sorption intensity); 1/*n* generally ranges between 0.7–1.0, indicating that sorption data are frequently slightly non-linear [56].

The Langmuir isotherm (Equation (3)):

(3) $$\frac{1}{c_{S}}=\frac{1}{K_{L}\cdot c_{\max}\cdot c_{W}}+\frac{1}{c_{\max}}$$

where *c_s_* is the content of test substance adsorbed onto soil at adsorption equilibrium (mg·kg^−1^); *c_w_* is the mass concentration of test substance in the aqueous phase at adsorption equilibrium (mg·L^−1^); *c*_max_ is the maximum sorption capacity of the sorbent (mg·kg^−1^); *K_L_* is the Langmuir constant (L·mg^−1^).

The Dubinin–Radushkevich isotherm (Equation (4)):

(4) $$\log c_{S}=\log q_{D}-2B_{D}R^{2}T^{2}\log\left(1+\frac{1}{c_{W}}\right)$$

where *c_s_* is the content of the test substance adsorbed onto soil at adsorption equilibrium (mg·kg^−1^); *q_D_* is the theoretical isotherm saturation capacity (mg·kg^−1^); *B_D_* is the Dubinin–Radushkevich isotherm constant related to adsorption energy (mol^2^·kJ^−2^); *R* is the gas constant (0.008314 kJ·mol^−1^·K^−1^); *T* is the absolute temperature (298 K); *c_w_* is the mass concentration of test substance in the aqueous phase at adsorption equilibrium (mg·L^−1^).

The mean free energy *E_D_* (kJ·mol^−1^) of adsorption per molecule of adsorbate was calculated according to Equation (5).

(5) $$E_{D}=\frac{1}{\sqrt{2B_{D}}}$$

where *E_D_* is the free energy (kJ·mol^−1^); *B_D_* is the isotherm constant (mol^2^·kJ^−2^).

The Temkin isotherm (Equation (6)):

(6) $$\theta =\frac{RT}{\Delta Q}\ln K_{0}+\frac{RT}{\Delta Q}\ln c_{W}$$

where *θ* is the fractional coverage (*c_s_*/*c*_max_ (mg·kg^−1^/mg·kg^−1^) value of *c*_max_ taken from the Langmuir equation; *K*_0_ is the equilibrium binding constant (L·mg^−1^); *R* is the gas constant (0.008314 kJ·mol^−1^·K^−1^); *T* is the absolute temperature (298 K); ∆*Q* is the variation of adsorption energy (kJ·mol^−1^) (∆*Q* = (−∆H)); *c_w_* is the mass concentration of test substance in the aqueous phase at adsorption equilibrium (mg·L^−1^).

#### 3.6.1. Determination of Sorption Coefficient in the Presence of Co-Contaminant

##### Investigation of the Heavy Metal Influence

The influence of heavy metals on the mobility of 5-FU and MTX was assessed on the example of cadmium. Each of the three tested soils was contaminated with cadmium at three concentration levels (0.4, 4.0, 40 mg·kg^−1^ dry mass (d.m.)) so that the content of a given pollutant in the sample was lower, equal, and exceeded the permitted concentration in soils from group B according to the Appendix to the Polish Directive of the Ministry of the Environment (Dz.U. 2002 nr 165 poz. 1359) [54]. For this purpose, an appropriate amount of a methanolic solution of cadmium chloride CdCl_2_ (at a concentration of 10 or 100 mg·L^−1^) was added to the weighed soils (1.00 g) and allowed to evaporate at room temperature, protected from light. Sample preparation and the actual test were performed as the standard test presented in Figure 6.

##### Sorption Investigation of ADs in the Presence of Metoprolol

Single point sorption coefficients (*K_d_*) for MTX and 5-FU were determined (10 mg·L^−1^ of MTX/5-FU) in the presence of MET (10 mg·L^−1^). In parallel, the samples containing only single compound were also prepared and subjected for further experiments. The standard stock solution of MET (100 mg·L^−1^) was prepared in 0.01 M CaCl_2_. The standard solutions of investigated anticancer drugs (10 mg·L^−1^) were prepared by dilution of the respective stock solution (1000 mg·L^−1^). In the case of MTX, the amount of DMSO in working solution did not exceed 1%.

### 3.7. Instrumental Analysis

The analytical system, Perkin Elmer Series 200, consisted of a chromatographic interface (Link 600), a binary pump, a UV/Vis detector, a vacuum degasser; additionally, a Rheodyne injection valve was used. All samples obtained during the sorption studies were analyzed in isocratic reversed phase mode using a Gemini C6-Phenyl column for 5-FU, MTX, and MET (110 Å, 5 µm, 150 mm × 4.6 mm), Phenomenex (Torrance, CA, USA). The analytical wavelengths of 266 nm, 302 nm, and 220 nm were used for 5-FU, MTX, and MET, respectively. For 5-FU, the mobile phase consisted of ACN:H_2_O (5:95, *v*/*v*) at a flow rate 0.7 mL·min^−1^. For MTX, the mobile phase consisted of ACN:H_2_O + 0.1% HCOOH (12:88, *v*/*v*) at a flow rate 0.7 mL·min^−1^. For MET, the previously published method was used. The mobile phase consisted of ACN:buffer H_2_O, 1 mM CH_3_COONH_4_ + 0.1% HCOOH at pH 3.56 (9:91) [36]. The injection volume of all applied methods was 50 μL. All chromatographic analyses were carried out on two replicates. The applied analytical methods were fully validated according to Konieczka et al. [57], and their selected metrological parameters are presented in Table 6.

## 4. Conclusions

This is the first examination of the sorption of 5-fluorouracil and methotrexate in soil. Investigated compounds have been considered to have slight or low mobility in the soil matrix (depending on soil), and their sorption strongly depends on environmental conditions. 5-fluorouracil may be more mobile in comparison to methotrexate; however, different correlations were observed between the investigated compounds and soil properties. For 5-FU, the highest sorption potential was observed in case of soil characterized by the highest OM content (G2, alluvial soil). On the other hand, for MTX the highest *K_d_* (12.94 L·kg^−1^) was determined for soil G4 (acid brown soil), characterized by the highest content of mineral fraction, moderate content of organic matter (OM = 7.7%), and the lowest pH (pH_KCl_ = 5.1) among tested soils. Based on the obtained results, it was also pointed out that in the case of ionic compounds it is crucial in the evaluation of their sorption potential to take into account their acid–base properties. Since during the sorption experiments 5-FU occurred mostly as neutral species, it is suspected that its sorption onto soil can be revealed from partitioning to neutral moieties of organic matter by, for example, π–π interactions.

However, in the case of soil G2 and G5, MTX occurred mostly as a molecule possessing one positive charge (protonated nitrogen in pteridine ring) and, due to dissociation of carboxylic groups, two negative charges. In the case of soil G4, approximately 50% of MTX molecules had one carboxylic group not dissociated (zwitterionic species), and the rest occurred as monoanionic species. Therefore, it might be suspected that MTX could interact with soil via electrostatic interactions, due to both positive and negative charge via cation–anion interactions or cation bridging.

Moreover, it was proved that the presence of other soil contaminants (such as heavy metals (Cd^2+^) or other pharmaceuticals (metoprolol, MET)) may strongly influence their mobility in soil structures. The investigated co-contaminant (MET) caused around a 25-fold increased sorption of 5-FU, whereas it caused diminished sorption of MTX. Therefore, the mobility of both compounds may differ significantly in environmental conditions because they would never exist there as single compounds. Moreover, the influence of environmental conditions such as pH and ionic strength on the sorption of 5-FU and MTX was clearly demonstrated.

## Figures and Tables

**Table 1:** Selected physico-chemical properties of the investigated compounds [33,35,36].

Compound [CAS Number] (Abbreviation)	Selected Physico-Chemical Properties	Structure
5-fluorouracil [51-21-8] (5-FU)	M = 130.1 g·mol^−1^ SH_2_O = 11,100 mg·L^−1^ LogK_ow_ = −0.89	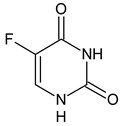
Methothrexate [59-05-2] (MTX)	M = 454.4 g·mol^−1^ SH_2_O = 2600 mg·L^−1^ LogK_ow_ = −1.85	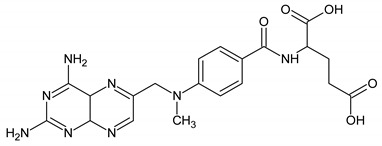
Metoprolol [56392-17-7] (MET)	M = 267.4 g·mol^−1^ SH_2_O = 16,900 mg·L^−1^ LogK_ow_ = 1.95	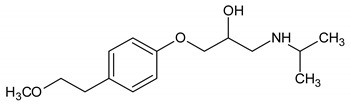

**Table 2:** Isotherm parameters of adsorption of 5-fluorouracil (5-FU) and methotrexate (MTX) onto soils G2 (alluvial soil), G4 (acid brown soil), and G5 (acid and leached brown soil).

Isotherm Model	Parameter	G2	G4	G5	G2	G4	G5
		5-FU			MTX		
Linear Isotherm	*K_d_* (L·kg^−1^)	6.36 ± 0.67	2.52 ± 0.36	2.98 ± 0.28	6.79 ± 0.74	12.94 ± 1.29	9.16 ± 0.93
	Isotherm equation	y = 6.357x + 34.802	y = 2.5175x + 23.115	y = 2.9804x + 15.622	y = 6.7861x + 38.179	y = 12.941x + 50.237	y = 9.1566x + 35.029
	*R* ^2^	0.928	0.875	0.940	0.914	0.926	0.924
Freundlich Isotherm	1/*n*	0.71	0.73	0.54	0.66	0.65	0.75
	*K_F_* (mg^1−1/n^·kg^−1^·L^1/n^)	21.44	9.73	15.69	30.56	53.60	28.42
	Isotherm equation	y = 0.7077x + 1.3312	y = 0.7372x + 0.9879	y = 0.5357x + 1.1956	y = 0.6619x + 1.4851	y = 0.6537x + 1.7292	y = 0.7468x + 1.4537
	*R* ^2^	0.921	0.879	0.848	0.996	0.997	0.996
Langmuir Isotherm	*c*_max_ (mg·kg^−1^)	149.26	41.49	19.92	112.36	126.58	217.39
	*K_L_* (L·mg^−1^)	0.19	0.35	10.68	0.52	1.07	0.19
	Isotherm equation	y = 0.0347x + 0.0067	y = 0.0684x + 0.0241	y = 0.0048x + 0.0502	y = 0.017x + 0.0089	y = 0.0074x + 0.0079	y = 0.0248x + 0.0046
	*R* ^2^	0.799	0.845	0.492	0.972	0.961	0.997
Dubinin–Radushkievich Isotherm	*B_D_* (mol^2^·kJ^−2^)	0.31	0.18	0.07	0.14	0.11	0.16
	*q_D_* (mg·kg^−1^)	250.38	82.72	57.53	176.63	252.81	204.46
	*E_D_* (kJ·mol^−1^)	1.27	1.65	2.61	1.92	2.13	1.76
	Isotherm equation	y = −3.7943x + 2.3986	y = −2.2431x + 1.9176	y = −0.9017x + 1.7599	y = −1.6658x + 2.2446	y = −1.3571x + 2.4028	y = −1.9822x + 2.3106
	*R* ^2^	0.700	0.739	0.513	0.888	0.903	0.895
Temkin Isotherm	Δ*Q* (kJ·mol^−1^)	3.61	1.25	0.79	9.90	7.37	7.85
	*K*_0_ (L·mg^−1^)	0.73	1.88	5.29	3.23	5.59	2.51
	Isotherm equation	y = 0.6868x − 0.2144	y = 1.9864x + 1.2529	y = 3.1225x + 5.2026	y = 0.2502x + 0.2935	y = 0.336x + 0.5784	y = 0.3158x + 0.2911
	*R* ^2^	0.855	0.696	0.648	0.856	0.827	0.829

**Table 3:** The influence of the presence of metoprolol (MET) on the sorption coefficients (K_d_* [L·kg^−1^]) of MTX and 5-FU onto investigated soils.

Tested Compound/s	MTX	MTX with MET	MET	MET with MTX
Soil G2	31.57 ± 1.88	23.86 ± 2.38	26.48 ± 7.64	9.90 ± 5.54
Soil G4	61.53 ± 3.62	74.91 ± 2.75	12.33 ± 1.02	5.47 ± 2.22
Soil G5	23.65 ± 1.00	17.33 ± 7.90	37.87 ± 4.85	11.89 ± 7.25
**Tested Compound/s**	**5 FU**	**5-FU with MET**	**MET**	**MET with 5-FU**
Soil G2	5.06 ± 1.24	22.09 ± 0.40	74.17 ± 2.78	182.75 ± 16.67
Soil G4	0.61 ± 0.21	1.72 ± 0.16	14.97 ± 1.27	10.55 ± 0.01
Soil G5	1.42 ± 0.34	37.55 ± 1.42	29.68 ± 0.46	10.47 ± 1.07

**Table 4:** Properties of soils used in this study.

Parameter	Soil G2 (Alluvial Soil)	Soil G4 (Acid Brown Soil)	Soil G5 (Acid and Leached Brown Soil)
pH_KCl_	7.1	5.1	5.8
OM [%]	18.6	7.7	4.1
CEC [cmol(+)·kg^−1^]	23.6	9.9	10.2
Colloidal Clay Fraction (<0.002 mm), [%]	14.0	5.0	12.0
SA [m^2^·kg^−1^]	1.6	0.9	10.9
ρ [g·cm^−3^]	1.1	1.3	1.4

CEC, cation exchange capacity; SA, surface area; ρ, density; OM, organic matter.

**Table 5:** Optimal S/L ratio and time of experiment.

Compound	Soil	S/L Ratio	Adsorption Equilibrium Time (h)
**5-FU**	G2	1:25	24
	G4	1:5	
	G5		
**MTX**	G2	1:15	24
	G4		
	G5		

**Table 6:** Validation parameters of the applied analytical methods using HPLC-UV/Vis.

Compound	*R* ^2^	Linearity Range (mg·L^−1^)	IQL (mg·L^−1^)	IDL (mg·L^−1^)	Precision (RSD) (%)	Accuracy (%)
**5-FU**	1.000 1.000	0.05–1.0 2.5–50.0	0.05	0.02	0.3–3.2	97.5–100.7
**MTX**	1.000 1.000	0.05–1.0 2.5–50.0	0.05	0.02	0.2–1.4	97.1–100.8
MET *	1.000	0.05–80	0.05	0.02	0.6–7.0	98.6–120.1

* The validation parameters determined by Maszkowska et al. [36].

## Data Availability

Most of the data generated or analysed during this study is included in this published article; if necessary, more data are available from the corresponding author on a reasonable request.

## Sample Availability

Samples of the compounds are not available from the authors.
